# Supplementary material for: Associations between infant and young child feeding practices and acute respiratory infection and diarrhoea in Ethiopia: A propensity score matching approach
Source: PLoS One. 2020 Apr 1;15(4):e0230978. doi: 10.1371/journal.pone.0230978 (PMC7112197; doi:10.1371/journal.pone.0230978)
Supplement: S1 Fig — (DOCX) [file pone.0230978.s001.docx]

| **Unmatched** | **Matched** |
| --- | --- |
| **ARI and early initiation of breastfeeding** | |
|  |  |
| **ARI and exclusive breastfeeding** | |
|  |  |
| **ARI and Predominant breastfeeding** | |
|  |  |
| **ARI and introduction of complementary foods** | |
|  |  |
| **ARI and continued breastfeeding at two years** | |
|  |  |
| **ARI and bottle feeding** | |
|  |  |

**ARI= Acute Respiratory Infection**

**S1 Fig. Distribution of propensity scores before and after nearest neighbour (0.1) matching in ARI and IYCF indicator**
