# Supplementary material for: Associations between infant and young child feeding practices and acute respiratory infection and diarrhoea in Ethiopia: A propensity score matching approach
Source: PLoS One. 2020 Apr 1;15(4):e0230978. doi: 10.1371/journal.pone.0230978 (PMC7112197; doi:10.1371/journal.pone.0230978)
Supplement: S2 Fig — (DOCX) [file pone.0230978.s002.docx]

| **Unmatched** | **Matched** |
| --- | --- |
| **Diarrhoea and early initiation of breastfeeding** | |
|  |  |
| **Diarrhoea and exclusive breastfeeding** | |
|  |  |
| **Diarrhoea and Predominant breastfeeding** | |
|  |  |
| **Diarrhoea and Introduction of complementary foods** | |
|  |  |
| **Diarrhoea and continued breastfeeding at two years** | |
|  |  |
| **Diarrhoea and bottle feeding** | |
|  |  |

**S2 Fig. Distribution of propensity scores before and after nearest neighbour (0.1) matching in diarrhoea and IYCF indicator**
