# Supplementary material for: Associations between infant and young child feeding practices and acute respiratory infection and diarrhoea in Ethiopia: A propensity score matching approach
Source: PLoS One. 2020 Apr 1;15(4):e0230978. doi: 10.1371/journal.pone.0230978 (PMC7112197; doi:10.1371/journal.pone.0230978)
Supplement: S1 Table — (DOCX) [file pone.0230978.s003.docx]

The association between infant and young child feeding, and acute respiratory infection and diarrhoea in Ethiopia, 2000 to 2016

| **IYCF factors** | **Acute respiratory infection** | | | **Diarrhoea** | |
| --- | --- | --- | --- | --- | --- |
|  |  | **Unadjusted** | **Adjusted** | **Unadjusted** | **Adjusted** |
|  | **n** | ***OR (95% CI)** | **^OR (95% CI)** | ***OR (95% CI)** | **^OR (95% CI)** |
| Early initiation of breastfeeding |  |  |  |  |  |
| No | 6517 | 1.00 | 1.00 | 1.00 | 1.00 |
| Yes | 8589 | 0.68 (0.61, 0.75) | 0.81 (0.72, 0.90) | 0.79 (0.73, 0.86) | 0.89 (0.78, 0.94) |
| Exclusive breastfeeding |  |  |  |  |  |
| No | 2106 | 1.00 | 1.00 | 1.00 | 1.00 |
| Yes | 2447 | 0.64 (0.52, 0.79) | 0.63 (0.50, 0.80) | 0.44 (0.36, 0.54) | 0.49 (0.39, 0.61) |
| Predominant breastfeeding |  |  |  |  |  |
| No | 1129 | 1.00 | 1.00 | 1.00 | 1.00 |
| Yes | 3424 | 0.77 (0.61, 0.98) | 0.80 (0.61, 1.04) | 0.57 (0.46, 0.69) | 0.58 (0.47, 0.73) |
| Introduction of complementary foods |  |  |  |  |  |
| No | 1204 | 1.00 | 1.00 | 1.00 | 1.00 |
| Yes | 1133 | 0.76 (0.58, 1.00) | 0.94 (0.68, 1.29) | 0.95 (0.78, 1.16) | 1.06 (0.84, 1.32) |
| Continued breastfeeding at 2 years |  |  |  |  |  |
| No | 402 | 1.00 | 1.00 | 1.00 | 1.00 |
| Yes | 1717 | 1.46 (1.05, 2.02) | 1.59 (1.04, 2.42) | 1.35 (1.05, 1.74) | 1.42 (1.05, 1.92) |
| Bottle feeding |  |  |  |  |  |
| No | 2182 | 1.00 | 1.00 | 1.00 | 1.00 |
| Yes | 1977 | 0.91 (0.78, 1.06) | 1.21 (1.03, 1.43) | 0.92 (0.82, 1.03) | 1.38 (1.07, 1.78) |

**n: weighted count of IYCF factors in unmatched data**

***indicates unadjusted ORs in unmatched data**

**^indicates adjusted ORs in unmatched data**
